# Book Notices

**Published:** 1882-01-20

**Authors:** 


### *BOOK NOTICES.*

THE OPIUM HABIT AND ALCOHOLISM. A Treatise on the Habits of Opium and its Compounds—Alcohol, Chloral Hydrate, Chloroform, Bromide Potassium, and Cannabis Indica—including their Therapeutical indications; with suggestions for treating various painful complications. By Dr. Fred. Heman Hubbard. A. S. Barnes & Co., New York. J. J. & S. P. Richards, Atlanta. In Cloth, 259 Pages. Price, $2.00.

It is a timely, able and interesting work, in which the habits above named, now so alarmingly prevalent in this country, are separately treated, and the abnormal conditions peculiar to each described, and the treatment best adapted to their relief fully detailed.

A MANUAL OF OPHTHALMIC PRACTICE. By Henry Schell, M. D., Surgeon to Wells' Eye Hospital, and Ophthalmic and Aural Surgeon to the Children's Hospital; with fifty-three illustrations. Philadelphia, D. G. Brinton. J. J. & S. P. Richards, Atlanta, Ga.

A neat volume of 263 pages. The various diseases of the eye with their surgical and medical treatment are ably and satisfactorily discussed. It will repay perusal, not only to the specialist, but to the general practitioner.

